# The Preparation of a Novel Hyperbranched Antifouling Material and Application in the Protection of Marine Concrete

**DOI:** 10.3390/ma15238402

**Published:** 2022-11-25

**Authors:** Junhao Xie, Shuai Qi, Qianping Ran, Lei Dong

**Affiliations:** 1 School of Material Science and Engineering, Southeast University, Nanjing 211189, China; 2 State Key Laboratory of High Performance Civil Engineering Materials, Jiangsu Research Institute of Building Science, Nanjing 211103, China

**Keywords:** hyperbranched polyglycerol, waterborne fluoro silicone polyurethane, graphene oxide, marine antifouling materials, marine concrete

## Abstract

Marine fouling on concrete has become one of the severest problems that damage the surface and even cause internal corrosion of marine concrete. Dissimilarly to the previous abuse of toxic antifoulants, developing hydrophobic waterborne antifouling materials could be regarded as one of the most environment-friendly and potential directions to protect marine concrete. However, the insufficient hydrophobicity, antifouling, and mechanical properties limit their application. Herein, we reported a series of hybrid coatings combining hyperbranched polyglycerol (HPG) decorated waterborne fluoro silicone polyurethane (H) and HPG-grafted graphene oxide (G-HPG) that improve the hydrophobicity, antifouling, and mechanical properties. The hybrid materials were modified by the hyperbranched polyglycerol synthesized based on the anionic-ring-opening reaction between glycerol and ethylene glycol or polyethylene glycol. Remarkably, the hydrophobicity (115.19°) and antifouling properties (BSA absorption of 2.33 μg/cm^2^ and *P. tricornutum* attachment of 1.289 × 10^4^ CFU/cm^2^) of the materials could be developed by the modification of HPG with higher generation numbers and backbone molecular weights. Moreover, the mechanical properties negligibly decreased (tensile strength decreased from 11.29 MPa to 10.49 MPa, same pencil hardness and adhesion grade as H of 2H and grade 2). The results revealed that the HPG of higher generation numbers and backbone molecular weights could benefit materials with enhanced antifouling properties and hydrophobicity. The method of hyperbranched modification can be regarded as potentially effective in developing the durability and antifouling properties of marine antifouling materials.

## 1. Introduction

Marine fouling on concrete, generally caused by the settlement and propagation of marine microorganisms on marine concrete buildings, such as algae and barnacles, has become one of the severest problems influencing the fuel consumption and the durability of marine concrete for the severe damage it causes on the surfaces and the resulting internal corrosion. Among the applications that concentrate on marine concrete antifouling, such as using toxic tributyltin (TBT) and copper salt, coating materials involving hydrophilic and hydrophobic materials are chosen more for their convenience, efficiency, and being environment-friendly [1,2]. Generally, while the hydrophilic materials, such as polyethylene glycol (PEG), could defend against biofoulings due to the produced hydration shell [3], the hydrophobic antifouling materials involving PDMS and fluorocarbon resin were used more for their better durability in a marine environment than hydrophilic materials and the exceptional ability to weaken the adhesion of biofouling [4]. Nevertheless, the weak mechanical properties, involving inadequate strength, hardness, and adhesion, made them prone to damage [5]. So far, many studies have been developed to improve the mechanical properties of hydrophobic antifouling materials. Tian et al. reported that proposing urethane groups into a diol with amide groups and flexible PDMS could significantly enhance the tensile strength [6]. Zhao et al. reported that the combination of epoxy and aminopropyl-terminated PDMS could endow coatings with excellent wear resistance [7]. Yang et al. reported that a novel material synthesized by monoglycidyl ether-terminated PDMS, epoxy resin, and dendritic polyamidoamine presented excellent wear resistance and adhesion [8]. However, those agents limited the further development of antifouling properties due to the decreasing hydrophobicity [9,10]. Meanwhile, the abuse of modifying agents [11], toxic solvents, and antifoulants [12,13] restricted the improvement of antifouling properties and disaccorded the demand for environmental protection. Therefore, the hydrophobic antifouling materials were supposed to be modified with a micro amount of high-efficiency and eco-friendly modifying agents and dispersed with nontoxic solvents.

Hyperbranched modifying agents possessed vast crosslinking sites and the ability to regulate the element distribution within coatings [14,15,16]. Mishra has verified that introducing hyperbranched polyether polyols could effectively enhance the hydrophobicity and mechanical properties effectively [17]. Unfortunately, they could not endow coatings with higher antifouling properties for their insignificant antifouling ability [18]. Since Frey developed the hyperbranched polyglycerol (HPG), presenting a similar hyperbranched structure to those hyperbranched polyester polyols, which was linked with ether bonds and capped with abundant hydroxyl groups, the extraordinary antifouling property of HPG has been reported [19]. To the best of our knowledge, HPG was not nearly used in modifying hydrophobic antifouling polymer coatings. The effect on antifouling concrete materials was a prospect to be realized.

Graphene oxide (GO) is one of the most effective nanomaterials to use in coating materials, solar cells, and many other fields [20]. GO can enhance the antifouling properties of materials for an increasing surface roughness, as well as hydrophobicity, with proper modifications [21], which consists of the introduction of rare earth oxides and other modified hydrophobic nanomaterials [22], and be an ideal antifoulant carrier to further enhance the antifouling properties of antifoulants [23]. Few studies have proven that generating HPG on GO sheets could be a significantly effective antifoulant in biomedical applications [24,25,26]. To the best of our knowledge, the enhancement of antifouling properties that combine GO grafting HPG with different molecular structures and antifouling polymer materials has not been reported. The antifouling reinforcement of materials introduced with the combination of GO and more varieties of HPG is a possible prospect.

Herein, considering improving the antifouling and mechanical properties and meeting the environmental demand, we report novel hybrid antifouling materials combined with HPG-modified hyperbranched waterborne fluoro silicone polyurethane, which contains a side chain of silane and is end-capped with 3, 3, 4, 4, 5, 5, 6, 6, 7, 7, 8, 8, 8-tridecaflfluoro-1-octanol [27], and a series of GO grafting different HPG categories. All categories of hyperbranched polyglycerol were synthesized by the anionic-ring-opening reaction of glycidol with ethylene glycol and PEG of different molecular weights. The results indicated that the variations of the properties could be easily regulated by introducing the GO grafting HPG with different generation numbers and the backbone molecular weights.

## 2. Materials and Methods

### 2.1. Materials

Unilateral dihydroxy siloxane 8822F2 (M_n_ ≈ 1400 g/mol) was supplied from Guangzhou Silok Polymer Co., Ltd. (Guangzhou, China), graphene oxide (GO) was purchased from Tanfeng Co., Ltd. (Suzhou, China), Phaeodactylum tricornutum Bohlin (*P. tricornutum*) was purchased from Shanghai Guangyu Co., Ltd. (Shanghai, China), and the other chemicals were all purchased from Aladdin Reagent. Ethylene glycol (EG); polyethylene glycol (PEG) (M_n_ = 600, 1000, and 2000 g/mol); and polyether polyol N220 (M_n_ ≈ 2000 g/mol) were vacuum-dehydrated at 110 °C for 2 h before use. Glycerol, 3, 3, 4, 4, 5, 5, 6, 6, 7, 7, 8, 8, 8-tridecaflfluoro-1-octanol (TFO), acetone, *N*,*N*-Dimethylformamide (DMF), trimethylamine (TEA), and 8822F2 were dehydrated with a 4 Å molecular sieve for at least three days. 2,2-bis (hydroxymethyl) propionic acid (DMPA) was vacuum-dried at 90 °C for 5 h before use. The GO was vacuum-dried at 40 °C for 24 h before use. 1-(3-dimethylaminopropyl)-3-ethylcarbodiimide hydrochloride (EDC), isophorone diisocyanate (IPDI), dibutyltin dilaurate (DBTDL), and potassium methoxide were directly utilized. The *P. tricornutum* culture solution was prepared by diluting the obtained f/2 of high concentration and algae concentrate into artificial seawater. The *P. tricornutum* was precultured at 20 °C for five days (light:dark = 12:12 h) in an illumination incubator and counted by a hemacytometer before the examination. The concentration of *P. tricornutum* in the antialgae examination was 10^6^ CFU/mL.

### 2.2. Methods of Synthesis and Preparation

#### 2.2.1. Synthesis of Hyperbranched Polyglycerol (HPG)

The synthesis process of HPG was modified by the anionic-ring-opening polymerization method reported by Frey [28]. Stoichiometric anhydrous EG and potassium methoxide were mixed into a 100-mL three-neck flask at 70 °C under mechanical stirring and then stirred in a vacuum for 30 min. Then, glycidol was gradually introduced into the flask for 15 h at a constant rate after being heated up to 155 °C under nitrogen protection. The reaction was terminated one hour after terminating the introduction of glycidol, and the yellowish product was collected without subsequent processing. The HPG was named after HPG-X-Y, where X represented the species of diols, and Y represented the generation number. The composition of HPG is presented in Table 1.

#### 2.2.2. Synthesis of Hyperbranched Waterborne Fluoro Silicone Polyurethane (H) Emulsion

The synthesis process of H materials was operated as follows. Since the materials introduced with HPG-EG-3 had relatively better mechanical and antifouling properties, we took the synthesis of H as an example. Anhydrous 26.4 g N220, 2.8 g DMPA, and 2.52 g 8822F2 were introduced into a 500 mL four-neck flask with a mechanical stirrer, condenser, nitrogen inlet, and outlet. When DMPA was utterly dissolved in the mixture, 14.89 g IPDI and two drops of DBTDL were slowly introduced into the flask, and the reaction proceeded at 90 °C for four hours. After the temperature was cooled to 60 °C, the chain extender EG mixed with a proper amount of acetone was introduced into the flask and maintained for another three hours. Subsequently, when heated to 85 °C, stoichiometric HPG-EG-3 dissolved in 10 mL DMF was introduced, and the moderate addition of acetone controlled the viscosity. After 3 h, 5.46 g TFO was added to the flask, and the reaction was continued for another 3 h. Afterward, 1.8 g neutralizer TEA was introduced into the flask as a neutralizer at room temperature, and the 30-min high-speed agitation was performed after DI water was poured into the flask. Finally, approximately 35% solid content emulsion was collected when the residual acetone was rotary evaporation removed. 

#### 2.2.3. Fabrication of HPG-Grafted GO (G-HPG)

HPG-modified GO (G-HPG) was fabricated by esterification between the carboxyl group on the surface of GO and the terminated hydroxyl group of HPG. GO (0.1 g) was initially ultrasonically dispersed in 100 mL DMF for 30 min. Then, the excessive amount of HPG and proper amount of EDC dissolved in 50 mL DMF was proposed in GO dispersion, and the mixture was ultrasonically dispersed for another 30 min. Afterward, the mixture was magnetically stirred at 40 °C for 24 h. After being washed with water and ethanol several times, the G-HPG products were obtained after vacuum drying at 40 °C for 24 h. The modified GO was named G-HPG-X-Y, where X represented the species of diols, and Y represented the generation number of grafted HPG. 

#### 2.2.4. Fabrication of the Hybrid Materials (H-G-HPG)

Hybrid materials were fabricated by the ultrasonic-dispersed method. Firstly, HPG-grafted GO was ultrasonically dispersed in 5 mL DI water for 30 min. Then, the HPG-grafted GO dispersion was mixed with H emulsion and continued dispersing for 30 min. The hybrid materials were obtained by spraying the same amount of emulsion on a tinplate. After the surface dried at room temperature and then dried at 50 °C for 12 h, the materials were obtained. The hybrid materials were named H-G-HPG-X-Y, where X represented the species of diols, and Y represented the generation number of grafted HPG. Meanwhile, materials proposed with different amounts of G-HPG-2000-3 were also fabricated.

### 2.3. Test Methods

#### 2.3.1. Chemical Structure

The approximate chemical structures of HPG, G-HPG, and H-G-HPG were characterized by a Nicolet iS20 Fourier-Transform Infrared Spectrometer (FTIR, Thermo Fisher, USA). Moreover, the accurate chemical structure, molecular weight (M_W_), polymer dispersity index (PDI), degree of branching (DB), and conversion rate of HPG were determined by LC-20ADXR Gel Permeation Chromatography (GPC, SHIMADZU, Kyoto, Japan), Agilent-1260-W High-Performance Liquid Chromatography (HPLC, Agilent, Santa Clara, CA, USA), and ^1^H-NMR and ^12^C-NMR utilizing AVANCE III HD 600 MHz Nuclear Magnetic Resonance (NMR, Bruker, Rheinstetten, Germany). 1H-NMR (DMSO-D6) δ (ppm): 4.0–4.5 (s, terminated OH), 3.40–4.0 (m, CH, CH_2_ in dendritic groups). ^12^C-NMR (DMSO-D6) δ (ppm): 63.0–64.0, 70.0–73.5 (L, linear groups); 78.0–80.5 (D, dendritic groups).

The interlamellar spacing between GO was evaluated by D8 ADVANCE X-ray Diffraction (XRD, Bruker, GER) and Cu Kα radiation. The spacing among GO or G-HPG sheets was calculated according to the Bragg equation:

(1) $$d=\frac{\lambda }{2sin\theta }$$

*d*—spacing among GO and G-HPG sheets (nm);
*λ*—0.15416 nm, the wavelength of X-ray radiation;
*θ*—diffraction angle of GO and G-HPG sheets (°).

#### 2.3.2. Hydrophobicity

The hydrophobicity of the materials was determined by measuring the water contact angle (WCA) value of 5 μL water droplets on the materials and calculated by the DSA255 contact angle tester (KRUSS, Hamburg, Germany).

#### 2.3.3. Mechanical Properties 

The tensile strength was measured by stretching the cast samples and performed at 100 mm/s on a WAW-1000 (Q) universal testing machine (Sunstest, Shenzhen, China) according to GB/T 528-2009. The hardness BEVS1301 pencil hardness tester (Shenghua, Beijing, China) determined the hardness according to GB/T 6739-2006. Furthermore, the adhesion was confirmed by the row lattice method using an adhesion tester (Moderner, Shanghai, China) according to GB/T 1720-1979.

#### 2.3.4. Surface Morphology

The microscopic surface morphology of the materials and G-HPG was evaluated and pictured by Quanta 250 scanning electron microscopy (SEM, FEI, Hillsboro, OR, USA) at an accelerating voltage of 5 KV. The samples were prepared by spray-coating on silicon wafers. Dimension ICON Atomic Force Microscopy (AFM, Bruker, GER) was used to investigate the detailed surface profiles, and the samples were prepared by spray-coating on glass slides. 

#### 2.3.5. Antifouling Properties 

The bovine serum albumin (BSA) absorption and antialgae experiments determined the antifouling properties. 

The BSA absorption test was performed by immersing five slices of each material (10 mm × 10 mm × 0.5 mm, coated on glass slides) into 5 mL 1 mg/mL BSA in PBS buffer solution (PH = 7.4) below 5 °C for 24 h. When the immersing process finished, the slices were removed, and the absorption values were determined at the absorbance of *λ* = 280 nm by the TU-1810spc Ultraviolet spectrophotometer (UV–Vis, PERSEE, Beijing, China). The quantitative relation between the residual concentration of BSA and the absorbance was fitted in Figure S1, and the equation of BSA absorption was as follows:

(2) $$A=0.65943C-0.00505\;\left(R^{2}=0.99\right)$$

A —Absorbance at *λ* = 280 nm;
- C—Concentration of BSA (mg/mL).

Then, the BSA absorption value was calculated by the following formula:

(3) $$Q=\frac{\left(C_{i}-C_{e}\right)V}{S}\times 1000$$

Q—BSA absorption per square centimeter (μg/cm^2^);
C_i_—Concentration of BSA before the test (mg/mL);
C_e_—Concentration of BSA after the test (mg/mL);
V—the volume of PBS solution of BSA (mL);
S—Area of each sample piece (cm^2^).

The antialgae examination was performed by immersing five slices of each material (R5 mm × 0.5 mm, coated on glass slides) into a 5 mL *P. tricornutum* culture solution at 20 °C for 24 h (light: dark = 12:12 h) in an illumination incubator. When the immersing process finished, the slices were removed, and the absorption values were determined at the absorbance of *λ* = 600 nm by the Ultraviolet spectrophotometer. The quantitative relation between the residual concentration of *P. tricornutum* and the absorbance was fitted in Figure S2, and the equation of *P. tricornutum* attachment was as follows:

(4) $$A=2.66082\times 10^{-7}C-0.00414\;\left(R^{2}=0.99\right)$$

A —Absorbance at *λ* = 600 nm;
- C—Concentration of *P. tricornutum* (CFU/mL).

(5) $$Q=\frac{C_{0}-C_{i}}{\pi R^{2}}\times V$$

*Q*—*P. tricornutum* attachment per square centimeter (CFU/cm^2^);
*C*_0_—Concentration of *P. tricornutum* before the test (mg/mL);
*C_i_*—Concentration of *P. tricornutum* after the test (mg/mL);
*V*—the volume of *P. tricornutum* culture solution (ml);
*R*—Radium of each sample piece (cm).

## 3. Results and Discussion

### 3.1. Characterizations of Hyperbranched Polyglycerol (HPG), HPG-Grafted GO (G-HPG), and Hybrid Materials (H-G-HPG)

**Figure 1:**
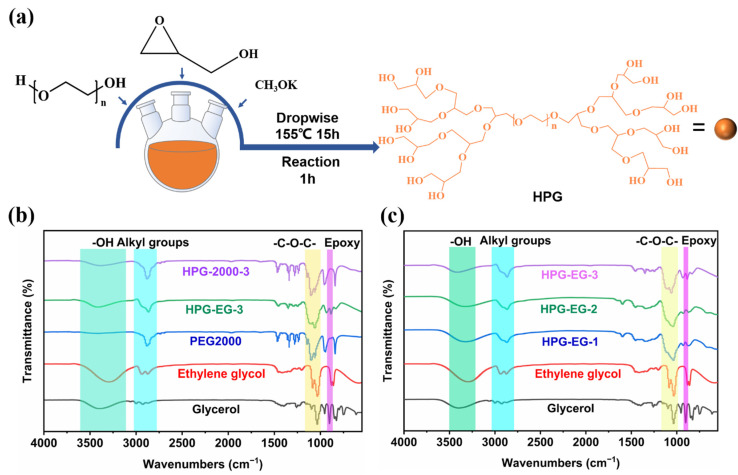
(**a**) Synthesis of HPG. (**b**) FTIR of glycerol, ethylene glycol, PEG2000, HPG-EG-3, and HPG-2000-3. (**c**) FTIR of glycerol, ethylene glycol, HPG-EG-1, HPG-EG-2, and HPG-EG-3.

As shown in Figure 1a, HPG was synthesized via the anionic-ring-opening polymerization method. Therefore, besides confirming M_W_ and PDI, the formation of the hyperbranched structure, the complete reaction of the epoxy group on glycerol, the formation of ether bonds at the dendritic units, and terminated hydroxyl groups were supposed to be identified. In Figure 1b,c, the peak at around 3400–3300 cm^−1^ was assigned to the stretching vibration of the branched hydroxyl groups (-OH). The peak at around 1000–1100 cm^−1^ corresponded to the stretching vibration of the ether bond (-C-O-C-). The peak of the ether bonds showed a delicate blue shift as the backbone molecular weight of HPG increased, which might be attributed to the weaker interaction among ether bonds in branched units and other HPG molecules formed as the backbone molecular weight of HPG increased. Moreover, the absence of peaks at 3050–3000 cm^−1^, 1725–1705 cm^−1^, and 840–750 cm^−1^ preliminarily confirmed the complete reaction of the epoxy group from glycerol. As shown in Figures S3 and S4, the complete reaction of the epoxy group was further confirmed by the disappearance of peaks at 2.5 ppm–2.6 ppm in ^1^H-NMR and the disappearance of peaks at the peak at 4.17 min in HPLC. Moreover, the degree of branching (DB) of HPG was calculated from the ^12^C-NMR in Figure S5 and demonstrated in Table 2. The DB of products was between 0.5 and 0.6, indicating the formation of the hyperbranched structure of HPG [28].

As shown in Table 2, the M_W_ of HPG-EG-1 to HPG-EG-3 varied from 532 to 1263, and the PDI ranged from 1.28 to 1.58, indicating the successful synthesis of multigeneration HPG with sprinkling by-products formed. Meanwhile, the M_W_ of HPG-EG-3 to HPG-2000-3 varied from 1263 to 3680, the PDI ranged from 1.09 to 1.60, and the position of the product peak did not coincide with that of glycerol at 4.17 min in HPLC shown in Figure S3, further indicating the successful synthesis of HPG of different backbone molecular weights with scattering by-products produced. As shown in Table 2, the M_W_ of HPG-EG-1 to HPG-EG-3 varied from 532 to 1263 and the PDI ranged from 1.28 to 1.58, indicating the successful synthesis of multigeneration HPG with sprinkling by-products formed. Meanwhile, the M_W_ of HPG-EG-3 to HPG-2000-3 varied from 1263 to 3680, the PDI ranged from 1.09 to 1.60, and the position of the product peak did not coincide with that of glycerol at 4.17 min in HPLC shown in Figure S3, further indicating the successful synthesis of HPG of different backbone molecular weights with scattering by-products produced. As shown in Table 2, the M_W_ of HPG-EG-1 to HPG-EG-3 varied from 532 to 1263, and the PDI ranged from 1.28 to 1.58, indicating the successful synthesis of multigeneration HPG with sprinkling by-products formed. Meanwhile, the M_W_ of HPG-EG-3 to HPG-2000-3 varied from 1263 to 3680, the PDI ranged from 1.09 to 1.60, and the position of the product peak did not coincide with that of glycerol at 4.17 min in HPLC shown in Figure S3, further indicating the successful synthesis of HPG of different backbone molecular weights with scattering by-products produced. 

**Figure 2:**
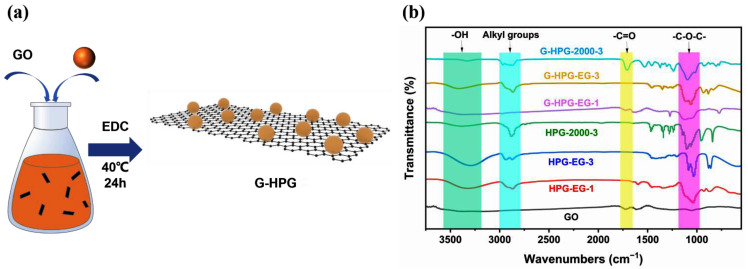
(**a**) Fabrication of G-HPG; (**b**) FTIR of G-HPG.

As demonstrated in Figure 2a, G-HPG was fabricated by esterifying the hydroxyl groups from HPG and the carboxyl groups from GO. Therefore, the alkyl and ether groups from HPG were indicated to prove the grafting of HPG on GO. As shown in Figure 2b, the peaks at around 2975 cm^−1^ and 2850 cm^−1^ among GO and G-HPG were referred to as the alkyl groups stretching vibration from HPG [24]. The peaks at 1127 cm^−1^ and 1057 cm^−1^ were more evident since the grafting of HPG, referring to the ether bonds stretching vibration from HPG. 

**Figure 3:**
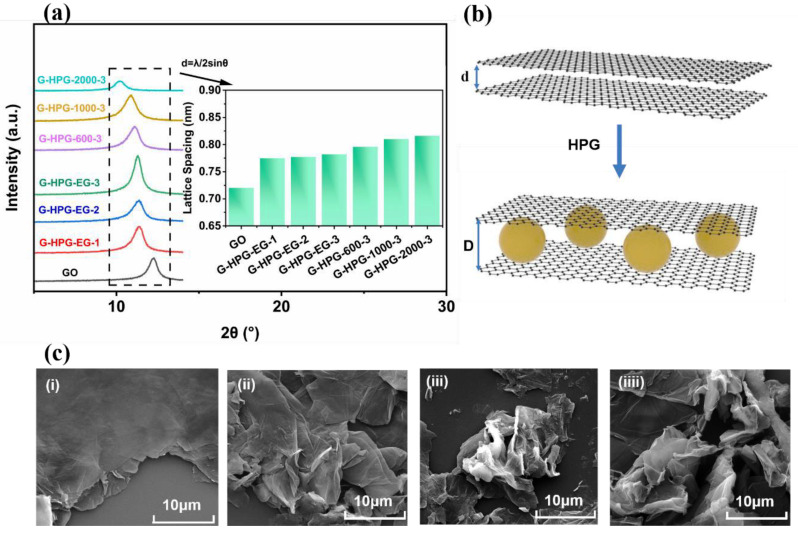
(**a**) XRD and calculated lattice spacing of G-HPG, (**b**) schematic explanation of the increasing lattice spacing among G-HPG sheets, (**ci**) SEM of GO, (**cii**) SEM of G-HPG-EG-1, (**ciii**) SEM of G-HPG-EG-3, and (**ciiii**) SEM of G-HPG-2000-3.

Additionally, the spacing among G-HPG sheets was mainly directed to the structural differences among the varieties of grafted GO. As shown in Figure 3a, the spacing between GO sheets can be calculated as 0.72 nm, where the 2θ value was at 2θ = 12.21° [16]. Moreover, the 2θ values of G-HPG diminished as the backbone molecular weight and generation number of HPG increased. The most significant spacing of 0.816 nm was performed by the grafting of HPG-2000-3, indicating the larger spacing between G-HPG sheets owing to the increase in the backbone molecular weight of HPG. As explained in Figure 3b, the increase in spacing between G-HPG sheets could be attributed to the more significant steric hindrance affected by the increase in the backbone molecular weight and generation number of grafted HPG. 

SEM was applied to confirm further the modification of the G-HPG surface and the spacing among G-HPG sheets. As shown in Figure 3(ci), GO presented sheets-like with significant thickness and smooth surfaces. Meanwhile, no noticeable wrinkle on the surface and the edge of GO sheets was found [29]. After grafting HPG on GO, as shown in Figure 3(cii–ciiii), the thick agglomerated GO sheets were separated into smaller pieces, and the wrinkles started appearing on the surface of G-HPG sheets, which indicated the grafting of HPG. As the generation number and backbone molecular weight of HPG increased, the separation of G-HPG sheets appeared increasingly apparent. It could result from the increasing molecular volume of grafted HPG and, hence, endowed more significant spacing with the G-HPG sheets. Dissimilarly, the variation of wrinkles amount within each species of G-HPG was not apparent. It can be explained that the HPG reacted with the mostly edge-enriched carboxyl groups of GO sheets, causing limited coverage on GO sheets.

**Figure 4:**
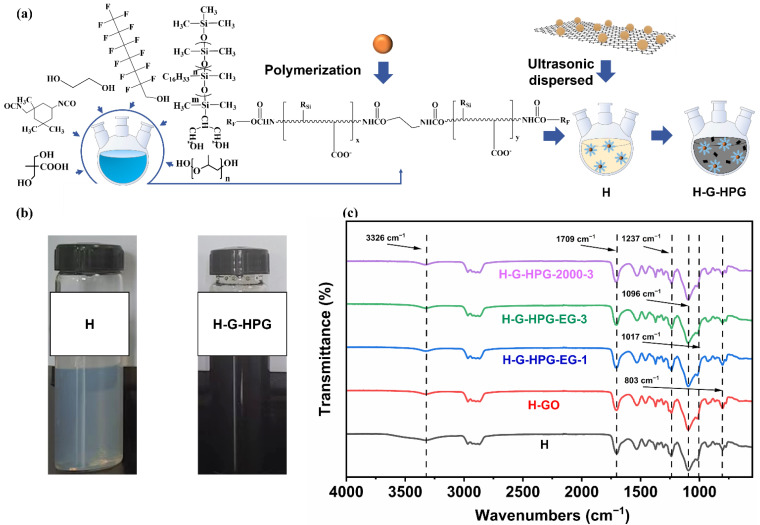
(**a**) Synthesis of H and fabrication of hybrid materials. (**b**) Image of H and H-G-HPG. (**c**) FTIR of polymer H and H-G-HPG materials.

As explained in Figure 4a, the synthesis of H was first performed by the reaction between isocyanate groups from IPDI and hydroxyl groups from HPG, 8822F2, TFO, and other diols. Moreover, the H-G-HPG hybrid emulsion was fabricated by ultrasonic dispersing the G-HPG into the H emulsion. Consequently, the forming of urethane groups and the disappearance of isocyanate groups were referred to as the extent of the synthesis of H and H-G-HPG. Figure 4b showed that the H materials appeared transparent and bluish with a yellowish tinge, while H-G-HPG appeared black. As demonstrated in Figure 4c, the peaks at 3326 cm^−1^ and 1709 cm^−1^ were assigned to the stretching vibration of the -N-H- bonds and C=O groups in the urethane group, and the disappearance at the peak of 2270 cm^−1^ confirmed the complete reaction of -NCO, which indicated the complete reaction of IPDI. Meanwhile, the peaks at 1017 cm^−1^ and 803 cm^−1^ correspond to the stretching of Si-O and Si-C of 8822F2, indicating the grafting of 8822F2 on the polyurethane chain. Moreover, the peak at 1237 cm^−1^ was assigned to the stretching vibration of -CF_3_ and demonstrated the grafting of TFO on the polyurethane chain [30]. Due to the low addition of GO and G-HPG, the difference in the chemical structure of H and H-G-HPG was nearly invisible within FTIR.

**Figure 5:**
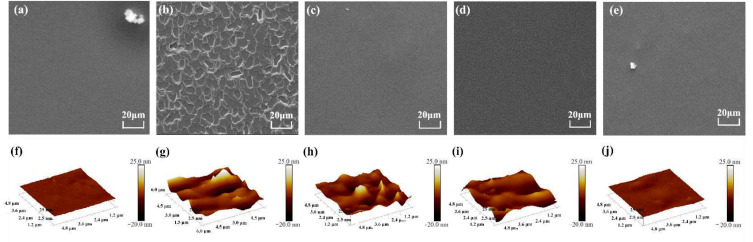
(**a**,**f**) Presented SEM and AFM morphology of H. (**b**,**g**) Presented SEM and AFM morphology of H-GO. (**c**,**h**) Presented SEM and AFM morphology of H-G-HPG-EG-1. (**d**,**i**) Presented SEM and AFM morphology of H-G-HPG-EG-3. (**e**,**j**) Presented SEM and AFM morphology of H-G-HPG-2000-3.

SEM was implemented to investigate the influence of different G-HPG on the surface morphology of H materials. As shown in Figure 5a, the surface of the H coating was smooth, attributed to the high concentration of soft segments. As demonstrated in Figure 5b, after the GO was proposed into H, the surface became rough owing to the phase separation caused by the strong interaction between GO sheets and polyurethane molecules. Nevertheless, as exhibited in Figure 5c–e, the surface became smooth again with the addition of G-HPG, though the difference between each coating was invisible in SEM. It can be interpreted that, besides increasing the proportion of soft segments, the grafted soft HPG obstructed the interaction between GO and polyurethane molecules and decreased the phase separation on the coating surface. 

AFM was applied to investigate the impact of different G-HPG on the surface morphology of H materials. As shown in Figure 5f, the surface morphology of H was smooth. In Figure 5g, the coating surface became rough after the introduction of GO, which was consistent with SEM. However, as explicitly shown in Figure 5h–j, the surface became smoother with G-HPG than with GO. When G-HPG-2000-3 was introduced, the coating surface became almost as smooth as the H materials. Of note, with the introduction of G-HPG-EG of lower generation number and backbone molecular weight, the roughness decreased insignificantly, in which the concentration of HPG did not significantly exceed GO. While the backbone molecular weight of grafting HPG further increased, the decline of surface roughness evolved significantly due to the hyper proportion of soft HPG than GO, which further diminished the interaction between GO sheets and H molecules, and endowed the smoother coating surface. 

### 3.2. Hydrophobicity of H-G-HPG

**Figure 6:**
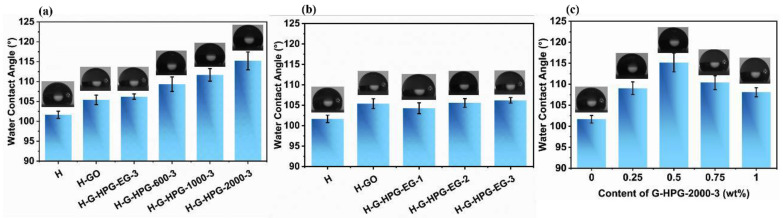
(**a**) WCA of H, H-GO, and materials introducing 0.5 wt% G-HPG grafting HPG with different backbone molecular weights. (**b**) WCA of H, H-GO, and materials introducing 0.5 wt% G-HPG grafting HPG-EG with different generation numbers. (**c**) WCA of materials introducing the different amount of G-HPG-2000-3.

The WCA value was implemented to confirm the variation of hydrophobicity among which materials proposed different varieties and proportions of G-HPG were endowed. As shown in Figure 6a, the WCA of H was valued at 101.67°. The WCA value of coating with the addition of 0.5 wt% GO raised to 105.38° due to the rough surface [31]. However, the increase was limited because of the hydrophilic groups on GO sheets. When proposing different varieties of G-HPG in the same generation number, the WCA value of materials further improved and, adding 0.5 wt% G-HPG-2000-3, reached the highest WCA value of 115.19°. This phenomenon could partly result in forming hydrophobic ester groups at the edges of G-HPG. Meanwhile, the relative hydrophobic center of the GO sheets was exposed since the HPG was enriched at the edges of GO, thus further improving the hydrophobicity of the materials. Moreover, in retrospect to the FTIR of HPG with different backbone molecular weights, the improved hydrophobicity of the materials might result in an agglomeration among the ether groups in dendritic units and backbone by the intramolecular hydrogen bonds [32]. With the agglomeration among ether bonds becoming significant, more hydrophobic alkyl groups of HPG were exposed to cover those agglomerated ether bonds and enhance the hydrophobicity further [33]. The contents of the ether groups on G-HPG increased with the backbone molecular weight of HPG, leading to more significant agglomeration. Therefore, the increase of hydrophobicity with the increase of the backbone molecular weight of HPG could be ascribed to the formation of ester groups, exposure of the hydrophobic center of GO sheets, and agglomeration among the ether groups. 

In Figure 6b, the WCA varies from coating proposing G-HPG grafting different generation numbers of HPG was demonstrated. When introducing G-HPG-EG-1, the WCA was slightly lower than H-GO because of the introduced hydrophilic ether bonds. The agglomeration of ether bonds was limited owing to the low generation number, and the hydrophilic effect of the hydroxyl groups was relatively more apparent, thus decreasing the hydrophobicity of the coating. When introducing G-HPG grafted with HPG of higher hyperbranched generation numbers, the hydrophobic effect that the agglomeration of ether bonds led to became more significant than the hydrophilic effect resulting in hydroxyl groups, hence enhancing the hydrophobicity of the coating. Owing to the limited increase in agglomeration, the development of hydrophobicity with the increasing generation numbers of HPG was not that conspicuous. As shown in Figure 6c, with different contents of G-HPG-2000-3, the WCA value of the materials varied and reached the highest value at the content of 0.5 wt%. The variation might primarily result in the proportion of the unreacted hydrophilic groups and grafted HPG on G-HPG sheets. With the content of G-HPG-2000-3 lower than 0.5 wt%, the content of unreacted hydrophilic groups on G-HPG was low, so the grafting HPG mainly influenced the hydrophobicity. When the content of G-HPG-2000-3 rose over 0.5 wt%, the unreacted hydrophilic groups were enough to be the main factor in decreasing the hydrophobicity. Therefore, the most appropriate content was 0.5 wt%.

### 3.3. Mechanical Properties of H-G-HPG

The mechanical properties of the materials were determined and compared by tensile strength, pencil hardness, and adhesion.

**Figure 7:**
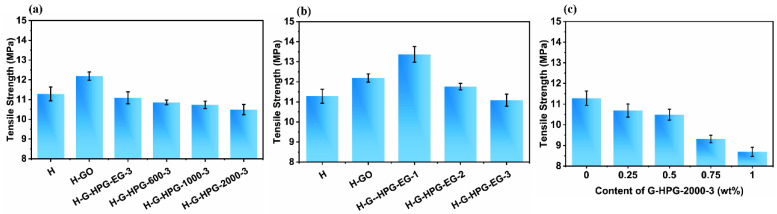
(**a**) Tensile strength of materials introducing G-HPG grafting HPG of different backbone molecular weights. (**b**) Tensile strength of materials introducing G-HPG grafting HPG-EG of different generation numbers. (**c**) Tensile strength varied from the content of G-HPG-2000-3.

As indicated in Figure 7a, the tensile strength of the coating raised from 11.29 MPa to 12.19 MPa as introduced GO due to the forceful interaction among the polar groups on the GO and H molecules. However, as G-HPG was introduced, the tensile strength of the coating decreased steadily and slightly with the increase of the backbone molecular weight of grafted HPG. The tensile strength decreased to 10.49 MPa with the introduction of 0.5 wt% G-HPG-2000-3. The decline of the tensile strength can be mainly attributed to the increasing content of soft HPG with the increase of the backbone molecular weight of HPG and decreasing concentration of GO with introducing the same concentration of G-HPG. Noticeably, the tensile strength of the worst-performing coating was about even with the original group, indicating that the introduction was viable. 

**Figure 8:**
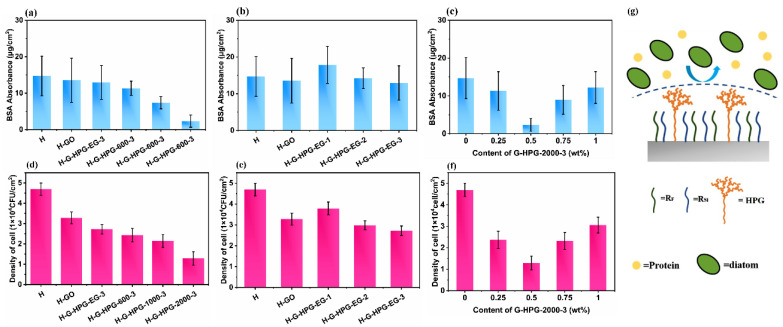
(**a**,**d**) Demonstrated the BSA absorption and P. tricornutum attachment of materials introducing G-HPG grafting HPG with different backbone molecular weights, (**b**,**e**) demonstrated the BSA absorption and *P. tricornutum* attachment of materials introducing G-HPG grafting HPG with different generation numbers, (**c**,**f**) demonstrated the BSA absorption and *P. tricornutum* attachment of materials introducing different content of G-HPG-2000-3, and (**g**) explained the antifouling mechanism of H-G-HPG materials.

As shown in Figure 7b, the tensile strength of the H-G-HPG-EG-1 coating raised to 13.36 MPa, higher than that of introducing GO. The increase in tensile strength of the coating might result in the formed strength-improving ester groups on H-G-HPG-EG-1, as well as the limited content of ether bonds on G-HPG-EG-1. The tensile strength declined when introducing G-HPG of higher generation numbers. The decline could be ascribed to the increasing content of soft hyperbranched ether bonds on G-HPG, while the proportion of GO decreased limitedly. As demonstrated in Figure 8c, with different contents of G-HPG-2000-3, the tensile strength of the coating decreased steadily and reached the lowest value of 8.69 MPa with the introduction of G-HPG-2000-3. The decline of the tensile strength could be attributed to the increasing content of grafted soft HPG on G-HPG, acting as the main reason causing the decline in tensile strength.

Simultaneously, the pencil hardness and row lattice methods were used to evaluate the hardness and adhesion of materials. As demonstrated in Table 3, the variation of pencil hardness grade and adhesion grade with the materials was semblable. The H-GO coating possessed relatively superior pencil hardness and adhesion grades among all the materials. As the generation number and backbone molecular weight of grafted HPG on G-HPG increased, the pencil hardness and adhesion grades declined, in which the grades of the worst-performing coating were similar to the H material.

The diminishment in pencil hardness could result in increasing content of the soft HPG segment and a decline in hydrogen bonding within materials. Furthermore, the decrease in adhesion grade indicated that the grafted HPG on G-HPG reduced the adhesive hydrogen bonding provided among oxygen functional groups and substrates, and the increasing content of HPG further decreased adhesion to substrates [31].

### 3.4. Antifouling Properties of H-G-HPG

The BSA absorption value could preliminarily define the antifouling ability of materials. As described in Figure 8a, the BSA absorption value of H-GO coating slightly decreased compared to H coating. The slight decrease could indicate that the developing hydrophobicity of H-GO coating could increase the antifouling property, but the rough surface might restrict the further enhancement of the antifouling property. When introducing 0.5 wt% G-HPG, the BSA absorption value declined continuously, and the magnitude of the decline became increasingly apparent with the development in the backbone molecular weight of grafted HPG. The lowest absorption value was reached at 2.33 μg/cm^2^ when proposing 0.5 wt% G-HPG-2000-3, suggesting the best antifouling anti-BSA ability. 

When G-HPG grafting HPG of different generations was introduced, as demonstrated in Figure 8b, the BSA absorption declined insignificantly with the increase in generation numbers. It could be ascribed that when introducing G-HPG grafting HPG of lower generation, the less steric hindrance and the deficiency of hydrophobicity restricted the resistance to BSA. In Figure 8c, when the content of G-HPG-2000-3 was below 0.5 wt%, the BSA absorption value gradually decreased, and the minimum reached 0.5 wt%. As the content of G-HPG-2000-3 exceeded 0.5 wt%, the BSA absorption value arose. This variation might be attributed to the apparent decline in hydrophobicity and steric hindrance. As a result, adding 0.5 wt% G-HPG-2000-3 in H coating performs relatively better BSA resistance. 

Antialgae experiments further confirmed the antifouling property of the materials. As shown in Figure 8d–f, the attachment capacity variation of *P. tricornutum*, which varied with the increase in the length of the backbone of HPG grafted on G-HPG, additive G-HPG grafted with different generations of HPG, and the content of additive G-HPG-2000-3 was consistent with the variation of BSA absorption. G-H-HPG-2000-3 demonstrated the lowest *P. tricornutum* attachment of 1.289 × 10^4^ CFU/cm^2^. In conclusion, BSA resistance and antialgae experiments determined that grafted HPG on G-HPG with higher hyperbranched generation and backbone molecular weight could invest materials with better antifouling properties. 

When HPG acts as an antifouling agent, one basis of antifouling is the hydration shell endowed by massive terminated hydrophilic hydroxyl groups. Furthermore, the steric hindrance on the coating surface, endued by the hyperbranched structure of HPG, performs the other essential factor defending the attachment of marine microorganisms. Obviously, since the utter consumption of terminated hydroxyl groups of HPG after grafting, the effect of the hydration shell could be negligible. Therefore, the steric hindrance performed primary cause of antifouling ability [4]. Considering the chemical structure of those diol-synthesized HPG, steric hindrance was mainly influenced by the backbone molecular weight and hyperbranched generation numbers of HPG. Besides the increase in steric hindrance, the increase in hydrophobicity of materials also enhanced the antifouling ability [34]. Therefore, the increase in the generation number and backbone molecular weight of HPG grafted on G-HPG, and the enhancement of hydrophobicity affected the antifouling properties.

## 4. Conclusions

In this study, the effect of hyperbranched polyglycerol with different generation numbers and backbone molecular weight improving hydrophobicity, mechanical and antifouling properties were investigated; the following conclusion can be drawn:

(1) The hydrophobicity of hybrid materials can be raised by introducing 0.5 wt% G-HPG graftings hyperbranched polyglycerol with higher generation numbers and backbone molecular weights for forming ester groups, exposure of the hydrophobic center of graphene oxide sheets, and increasing agglomeration among the ether groups. The highest water contact angle of 115.19° could be reached by introducing 0.5 wt% G-HPG-2000-3 in H material.
(2) The mechanical properties of hybrid materials slightly decreased by introducing 0.5 wt% G-HPG graftings hyperbranched polyglycerol with higher generation numbers and backbone molecular weights for the increasing amount of soft hyperbranched polyglycerol segments and decreasing amount of strengthening graphene oxide. The tensile strength of H-G-HPG-2000-3 decreased from 11.29 MPa to 10.49 MPa, and the pencil hardness and adhesion grade of H-G-HPG-2000-3 was same as H of 2H and grade 2
(3) The antifouling properties of hybrid materials can be significantly raised by introducing 0.5 wt% G-HPG graftings hyperbranched polyglycerol with higher generation numbers and backbone molecular weights for the increase in hydrophobicity and the more significant steric hindrance among hybrid materials and biofoulings. The lowest BSA absorption of 2.33 μg/cm^2^ and *P. tricornutum* attachment of 1.289 × 10^4^ CFU/cm^2^ could be reached by introducing 0.5 wt% G-HPG-2000-3 in H materials.

## Figures and Tables

**Table 1:** HPG designation and composition.

Sample	EG (mol)	PEG600 (mol)	PEG1000 (mol)	PEG2000 (mol)	Glycerol (mol)
HPG-EG-1	0.1	-	-	-	0.2
HPG-EG-2	0.1	-	-	-	0.6
HPG-EG-3	0.1	-	-	-	1.4
HPG-600-3	-	0.1	-	-	1.4
HPG-1000-3	-	-	0.1	-	1.4
HPG-2000-3	-	-	-	0.1	1.4

**Table 2:** Molecular weight, PDI, and degree of branching of HPG.

Samples	M_n_^a^	M_W_^a^	PDI ^b^	DB ^c^
HPG-EG-1	415	532	1.28	/
HPG-EG-2	578	931	1.61	/
HPG-EG-3	794	1263	1.59	0.58
HPG-600-3	1113	1759	1.58	0.54
HPG-1000-3	1621	2594	1.60	0.53
HPG-2000-3	3376	3680	1.09	0.50

^a^ Indicated by GPC. ^b^ Calculated by M_W/_M_n_. ^c^ calculated by DB = 2D/(2D + L) in ^12^C-NMR.

**Table 3:** Pencil hardness and adhesion of materials.

Sample	Pencil Hardness	Adhesion
H	2H	2
H-GO	3H	0
H-G-HPG-EG-1	2H	0
H-G-HPG-EG-2	2H	1
H-G-HPG-EG-3	2H	1
H-G-HPG-600-3	2H	1
H-G-HPG-1000-3	2H	2
H-G-HPG-2000-3	2H	2

## Data Availability

Not applicable.

## Supplementary Materials

The following supporting information can be downloaded at: https://www.mdpi.com/article/10.3390/ma15238402/s1: Figure S1: Fitting curve of the relationship between BSA concentration and absorbance; Figure S2: Fitting curve of the relationship between *P. tricornutum* cell concentration and absorbance; Figure S3: HPLC curves of HPG; Figure S4: ^1^H-NMR of HPG; Figure S5: ^12^C-NMR of HPG.
