# Supplementary material for: The Preparation of a Novel Hyperbranched Antifouling Material and Application in the Protection of Marine Concrete
Source: Materials (Basel). 2022 Nov 25;15(23):8402. doi: 10.3390/ma15238402 (PMC9741258; doi:10.3390/ma15238402)
Supplement: Supplementary file 1 [file materials-15-08402-s001.zip › materials-2045722-supplementary.pdf]

Supporting Information

The Preparation of a Novel Hyperbranched Antifouling Material and Application in the Protection of Marine Concrete

Junhao Xie [1,†], Shuai Qi [2,*,†], Qianping Ran [1,*], Lei Dong [1]

Figure S1 Fitting curve of the relationship between BSA concentration and absorbance.

Figure S2 Fitting curve of the relationship between P. tricornutum cell concentration and absorbance

Figure S3 HPLC curves of HPG

Figure S4 ^{1}H-NMR of HPG

Figure S5 ^{12}C-NMR of HPG

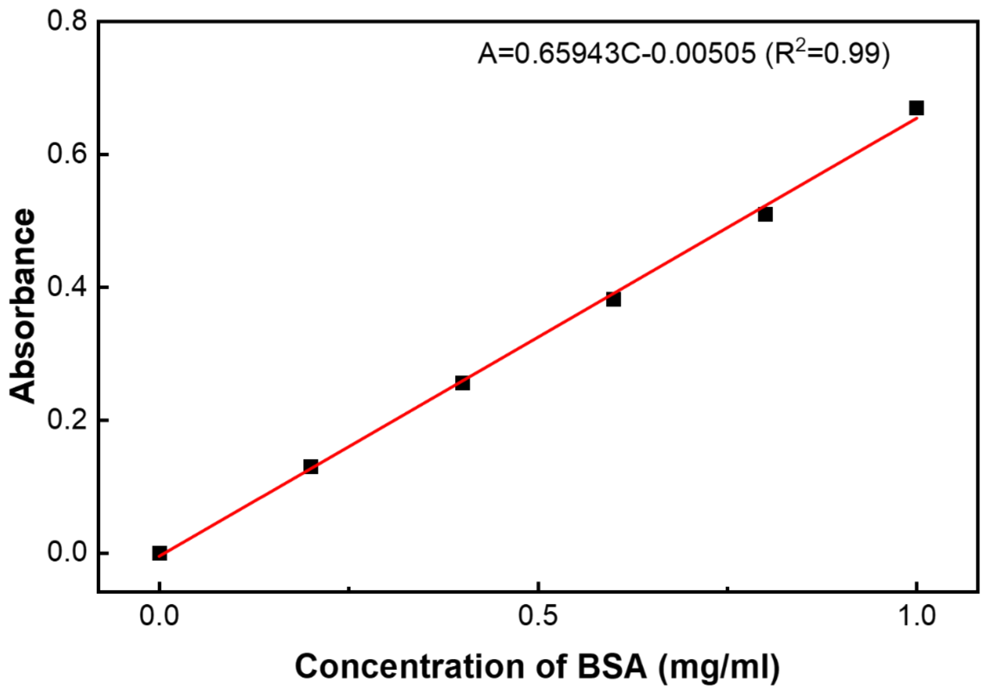

Figure S1 Fitting curve of the relationship between BSA concentration and absorbance

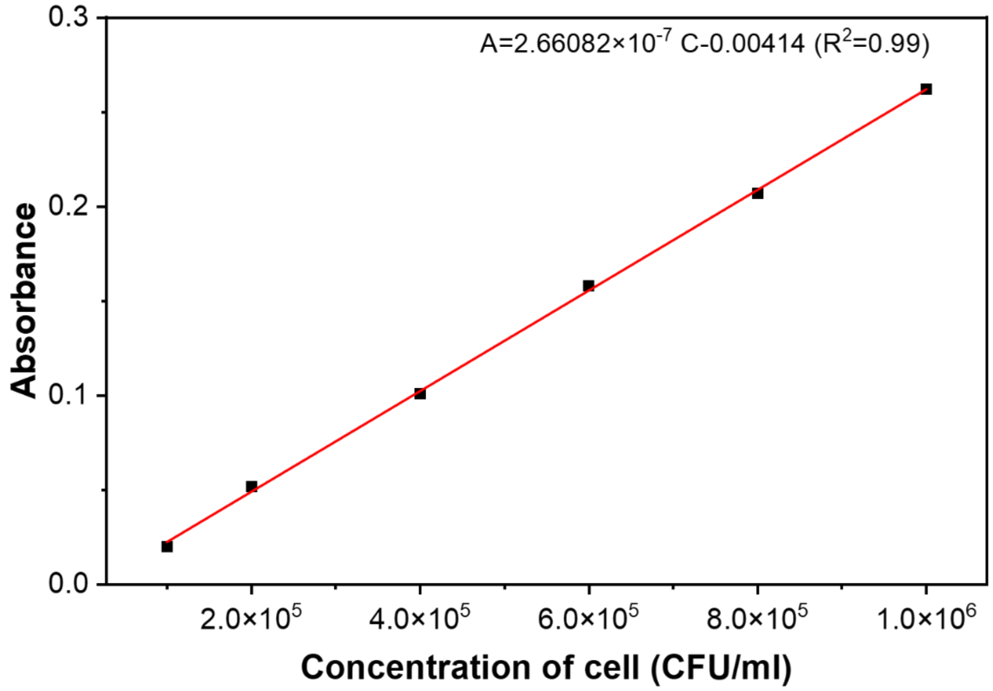

Figure S2 Fitting curve of the relationship between cell concentration and absorbance of P. tricornutum

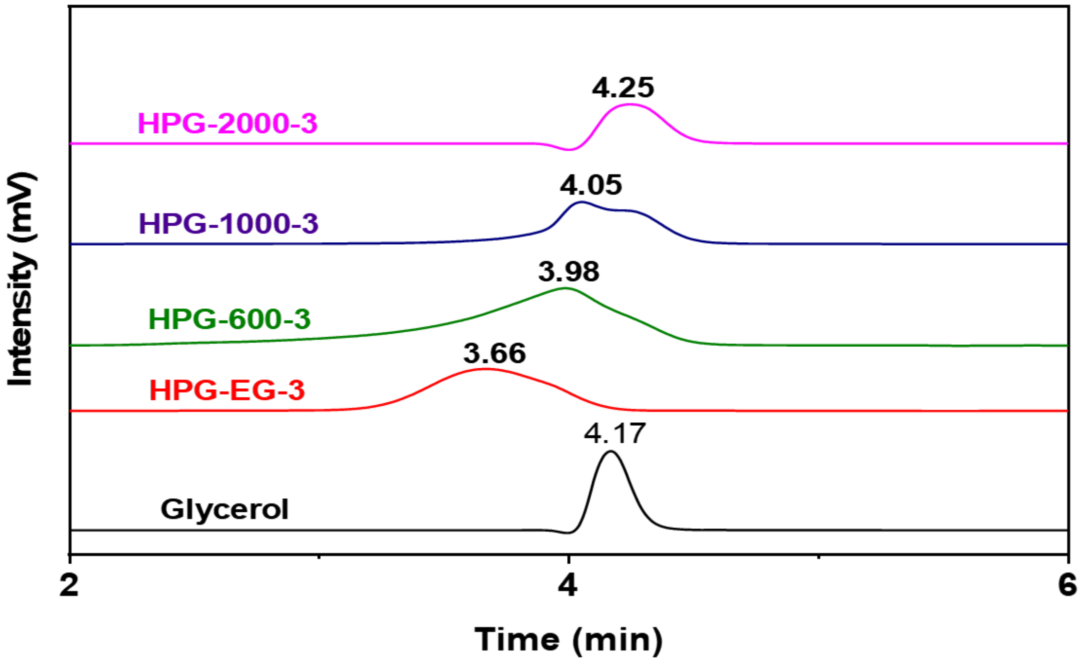

Figure S3 HPLC curves of HPG

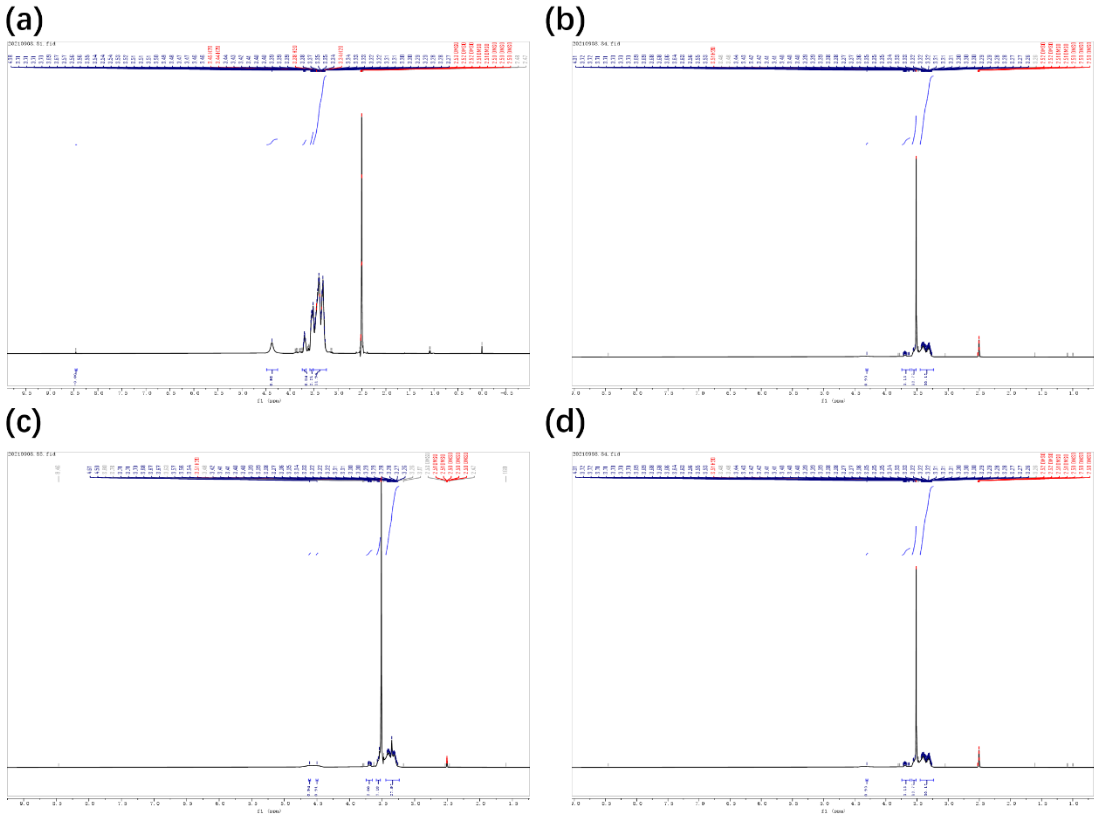

Figure S4 ^{1}H-NMR of HPG: (a) HPG-EG-3; (b) HPG-600-3; (c) HPG-1000-3; (d) HPG-2000-3

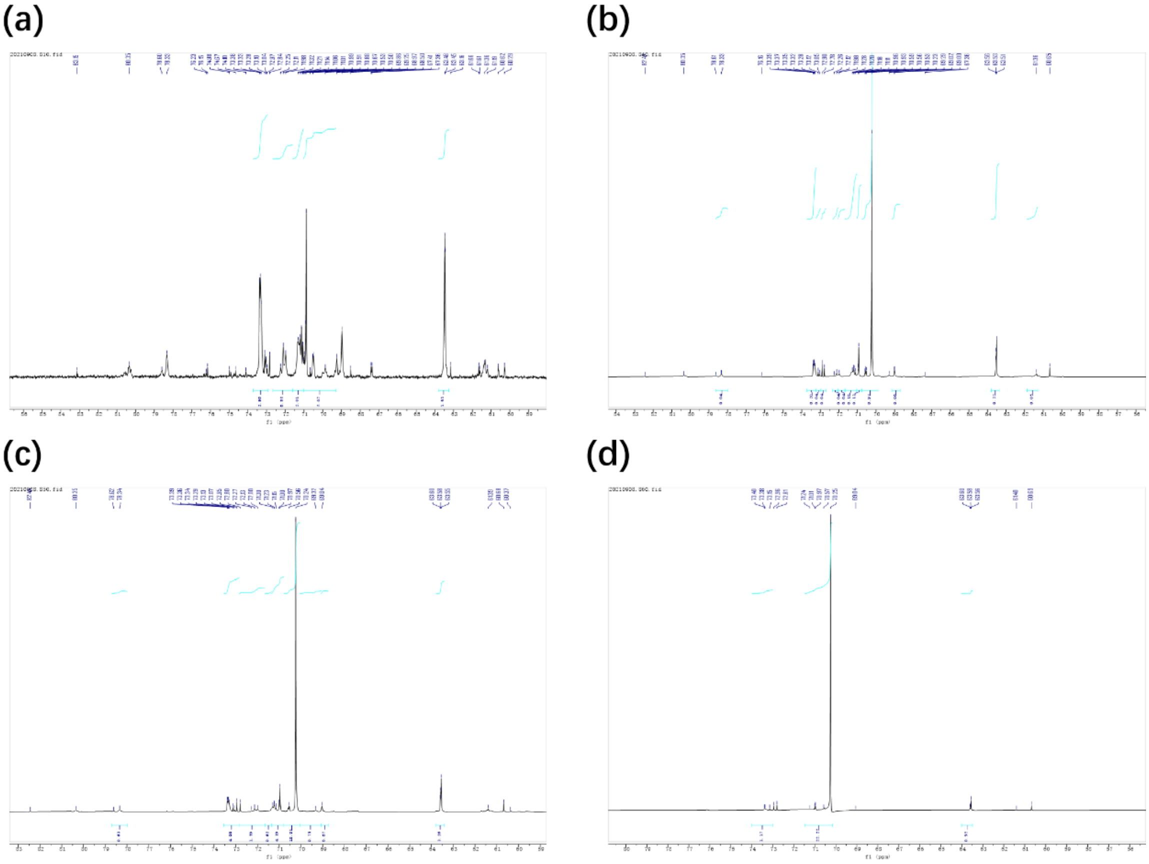

Figure S5 ^{12}C-NMR of HPG: (a) HPG-EG-3; (b) HPG-600-3; (c) HPG-1000-3; (d) HPG-2000-3
